# Supplementary material for: An Intranasal OMV-Based Vaccine Induces High Mucosal and Systemic Protecting Immunity Against a SARS-CoV-2 Infection
Source: Front Immunol. 2021 Dec 17;12:781280. doi: 10.3389/fimmu.2021.781280 (PMC8721663; doi:10.3389/fimmu.2021.781280)
Supplement: Supplementary file 1 [file DataSheet_1.docx]

**Supplementary material**

**A**

Hexapro MFVFLVLLPLVSSQCVNLTTRTQLPPAYTNSFTRGVYYPDKVFRSSVLHSTQDLFLPFFS 60

Spike MFVFLVLLPLVSSQCVNLTTRTQLPPAYTNSFTRGVYYPDKVFRSSVLHSTQDLFLPFFS 60

mC-Spike MFVFLVLLPLVSSQCVNLTTRTQLPPAYTNSFTRGVYYPDKVFRSSVLHSTQDLFLPFFS 60

************************************************************

Hexapro NVTWFHAIHVSGTNGTKRFDNPVLPFNDGVYFASTEKSNIIRGWIFGTTLDSKTQSLLIV 120

Spike NVTWFHAIHVSGTNGTKRFDNPVLPFNDGVYFASTEKSNIIRGWIFGTTLDSKTQSLLIV 120

mC-Spike NVTWFHAIHVSGTNGTKRFDNPVLPFNDGVYFASTEKSNIIRGWIFGTTLDSKTQSLLIV 120

************************************************************

Hexapro NNATNVVIKVCEFQFCNDPFLGVYYHKNNKSWMESEFRVYSSANNCTFEYVSQPFLMDLE 180

Spike NNATNVVIKVCEFQFCNDPFLGVYYHKNNKSWMESEFRVYSSANNCTFEYVSQPFLMDLE 180

mC-Spike NNATNVVIKVCEFQFCNDPFLGVYYHKNNKSWMESEFRVYSSANNCTFEYVSQPFLMDLE 180

************************************************************

Hexapro GKQGNFKNLREFVFKNIDGYFKIYSKHTPINLVRDLPQGFSALEPLVDLPIGINITRFQT 240

Spike GKQGNFKNLREFVFKNIDGYFKIYSKHTPINLVRDLPQGFSALEPLVDLPIGINITRFQT 240

mC-Spike GKQGNFKNLREFVFKNIDGYFKIYSKHTPINLVRDLPQGFSALEPLVDLPIGINITRFQT 240

************************************************************

Hexapro LLALHRSYLTPGDSSSGWTAGAAAYYVGYLQPRTFLLKYNENGTITDAVDCALDPLSETK 300

Spike LLALHRSYLTPGDSSSGWTAGAAAYYVGYLQPRTFLLKYNENGTITDAVDCALDPLSETK 300

mC-Spike LLALHRSYLTPGDSSSGWTAGAAAYYVGYLQPRTFLLKYNENGTITDAVDCALDPLSETK 300

************************************************************

Hexapro CTLKSFTVEKGIYQTSNFRVQPTESIVRFPNITNLCPFGEVFNATRFASVYAWNRKRISN 360

Spike CTLKSFTVEKGIYQTSNFRVQPTESIVRFPNITNLCPFGEVFNATRFASVYAWNRKRISN 360

mC-Spike CTLKSFTVEKGIYQTSNFRVQPTESIVRFPNITNLCPFGEVFNATRFASVYAWNRKRISN 360

************************************************************

Hexapro CVADYSVLYNSASFSTFKCYGVSPTKLNDLCFTNVYADSFVIRGDEVRQIAPGQTGKIAD 420

Spike CVADYSVLYNSASFSTFKCYGVSPTKLNDLCFTNVYADSFVIRGDEVRQIAPGQTGKIAD 420

mC-Spike CVADYSVLYNSASFSTFKCYGVSPTKLNDLCFTNVYADSFVIRGDEVRQIAPGQTGKIAD 420

************************************************************

Hexapro YNYKLPDDFTGCVIAWNSNNLDSKVGGNYNYLYRLFRKSNLKPFERDISTEIYQAGSTPC 480

Spike YNYKLPDDFTGCVIAWNSNNLDSKVGGNYNYLYRLFRKSNLKPFERDISTEIYQAGSTPC 480

mC-Spike YNYKLPDDFTGCVIAWNSNNLDSKVGGNYNYLYRLFRKSNLKPFERDISTEIYQAGSTPC 480

************************************************************

Hexapro NGVEGFNCYFPLQSYGFQPTNGVGYQPYRVVVLSFELLHAPATVCGPKKSTNLVKNKCVN 540

Spike NGVEGFNCYFPLQSYGFQPTNGVGYQPYRVVVLSFELLHAPATVCGPKKSTNLVKNKCVN 540

mC-Spike NGVEGFNCYFPLQSYGFQPTNGVGYQPYRVVVLSFELLHAPATVCGPKKSTNLVKNKCVN 540

************************************************************

Hexapro FNFNGLTGTGVLTESNKKFLPFQQFGRDIADTTDAVRDPQTLEILDITPCSFGGVSVITP 600

Spike FNFNGLTGTGVLTESNKKFLPFQQFGRDIADTTDAVRDPQTLEILDITPCSFGGVSVITP 600

mC-Spike FNFNGLTGTGVLTESNKKFLPFQQFGRDIADTTDAVRDPQTLEILDITPCSFGGVSVITP 600

************************************************************

Hexapro GTNTSNQVAVLYQDVNCTEVPVAIHADQLTPTWRVYSTGSNVFQTRAGCLIGAEHVNNSY 660

Spike GTNTSNQVAVLYQGVNCTEVPVAIHADQLTPTWRVYSTGSNVFQTRAGCLIGAEHVNNSY 660

mC-Spike GTNTSNQVAVLYQGVNCTEVPVAIHADQLTPTWRVYSTGSNVFQTRAGCLIGAEHVNNSY 660

*************.**********************************************

Hexapro ECDIPIGAGICASYQTQTNSPGSASSVASQSIIAYTMSLGAENSVAYSNNSIAIPTNFTI 720

Spike ECDIPIGAGICASYQTQTNSPGSASSVASQSIIAYTMSLGAENSVAYSNNSIAIPTNFTI 720

mC-Spike ECDIPIGAGICASYQTQTNSPGSASSVASQSIIAYTMSLGAENSVAYSNNSIAIPTNFTI 720

************************************************************

Hexapro SVTTEILPVSMTKTSVDCTMYICGDSTECSNLLLQYGSFCTQLNRALTGIAVEQDKNTQE 780

Spike SVTTEILPVSMTKTSVDCTMYICGDSTECSNLLLQYGSFCTQLNRALTGIAVEQDKNTQE 780

mC-Spike SVTTEILPVSMTKTSVDCTMYICGDSTECSNLLLQYGSFCTQLNRALTGIAVEQDKNTQE 780

************************************************************

Hexapro VFAQVKQIYKTPPIKDFGGFNFSQILPDPSKPSKRSPIEDLLFNKVTLADAGFIKQYGDC 840

Spike VFAQVKQIYKTPPIKDFGGFNFSQILPDPSKPSKRSPIEDLLFNKVTLADAGFIKQYGDC 840

mC-Spike VFAQVKQIYKTPPIKDFGGFNFSQILPDPSKPSKRSPIEDLLFNKVTLADAGFIKQYGDC 840

************************************************************

Hexapro LGDIAARDLICAQKFNGLTVLPPLLTDEMIAQYTSALLAGTITSGWTFGAGPALQIPFPM 900

Spike LGDIAARDLICAQKFNGLTVLPPLLTDEMIAQYTSALLAGTITSGWTFGAGPALQIPFPM 900

mC-Spike LGDIAARDLICAQKFNGLTVLPPLLTDEMIAQYTSALLAGTITSGWTFGAGPALQIPFPM 900

************************************************************

Hexapro QMAYRFNGIGVTQNVLYENQKLIANQFNSAIGKIQDSLSSTPSALGKLQDVVNQNAQALN 960

Spike QMAYRFNGIGVTQNVLYENQKLIANQFNSAIGKIQDSLSSTPSALGKLQDVVNQNAQALN 960

mC-Spike QMAYRFNGIGVTQNVLYENQKLIANQFNSAIGKIQDSLSSTPSALGKLQDVVNQNAQALN 960

************************************************************

Hexapro TLVKQLSSNFGAISSVLNDILSRLDPPEAEVQIDRLITGRLQSLQTYVTQQLIRAAEIRA 1020

Spike TLVKQLSSNFGAISSVLNDILSRLDPPEAEVQIDRLITGRLQSLQTYVTQQLIRAAEIRA 1020

mC-Spike TLVKQLSSNFGAISSVLNDILSRLDPPEAEVQIDRLITGRLQSLQTYVTQQLIRAAEIRA 1020

************************************************************

Hexapro SANLAATKMSECVLGQSKRVDFCGKGYHLMSFPQSAPHGVVFLHVTYVPAQEKNFTTAPA 1080

Spike SANLAATKMSECVLGQSKRVDFCGKGYHLMSFPQSAPHGVVFLHVTYVPAQEKNFTTAPA 1080

mC-Spike SANLAATKMSECVLGQSKRVDFCGKGYHLMSFPQSAPHGVVFLHVTYVPAQEKNFTTAPA 1080

************************************************************

Hexapro ICHDGKAHFPREGVFVSNGTHWFVTQRNFYEPQIITTDNTFVSGNCDVVIGIVNNTVYDP 1140

Spike ICHDGKAHFPREGVFVSNGTHWFVTQRNFYEPQIITTDNTFVSGNCDVVIGIVNNTVYDP 1140

mC-Spike ICHDGKAHFPREGVFVSNGTHWFVTQRNFYEPQIITTDNTFVSGNCDVVIGIVNNTVYDP 1140

************************************************************

Hexapro LQPELDSFKEELDKYFKNHTSPDVDLGDISGINASVVNIQKEIDRLNEVAKNLNESLIDL 1200

Spike LQPELDSFKEELDKYFKNHTSPDVDLGDISGINASVVNIQKEIDRLNEVAKNLNESLIDL 1200

mC-Spike LQPELDSFKEELDKYFKNHTSPDVDLGDISGINASVVNIQKEIDRLNEVAKNLNESLIDL 1200

************************************************************

Hexapro QELGKYEQGSGYIPEAPRDGQAYVRKDGEWVLLSTFLGRSLEVLFQGPGHHHHHHHHSAW 1260

Spike QELGKYEQGSGYIPEAPRDGQAYVRKDGEWVLLSTFLGRSLEVLFQGPGHHHHHHHHSAW 1260

mC-Spike QELGKYEQGSGYIPEAPRDGQAYVRKDGEWVLLSTFLGRSLEVLFQGPGHHHHHHHHSAW 1260

************************************************************

Hexapro SHPQFEKGGGSGGGGSGGSAWSHPQFEK-------------------------------- 1288

Spike SHPQFEKGGGSG-GGSGGSAWSHPQFEK-------------------------------- 1287

mC-Spike SHPQFEKGGGSG-GGSGGSAWSHPQFEKGGGSGGGSGGGSGLLRKGGEKIGEKLKKIGQK 1319

************ ***************

Hexapro -------------- 1288

Spike -------------- 1287

mC-Spike IKNFFQKLVPQPEQ 1333


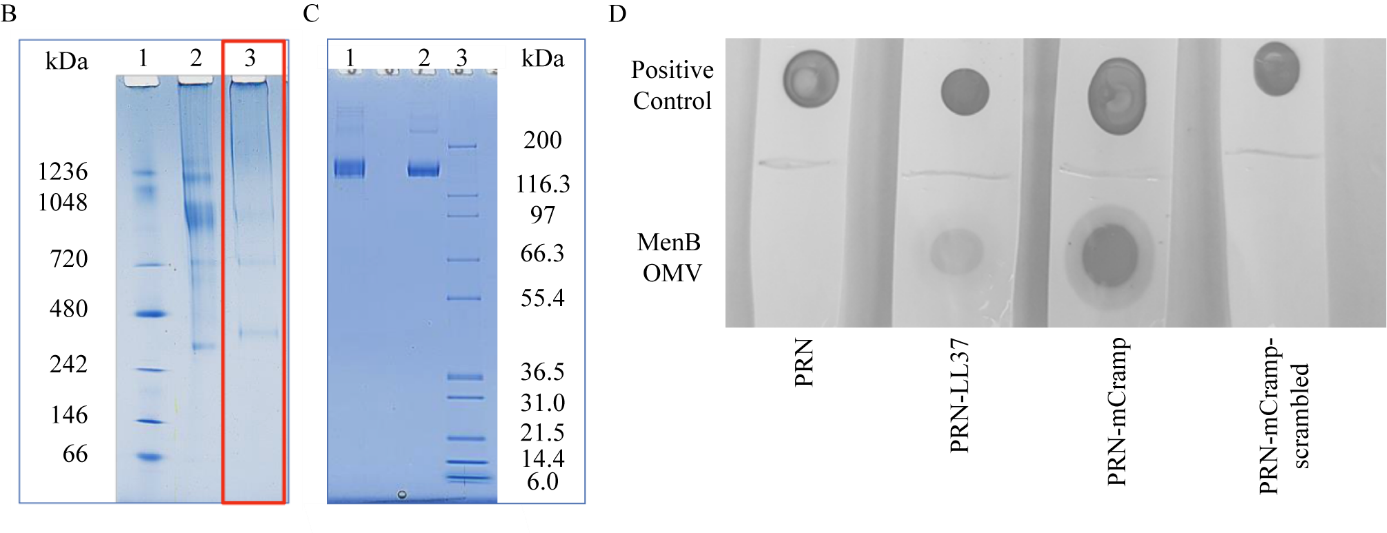


**Figure S1.** (**A**) Multiple sequence alignment of the Spike protein sequences. HexaPro spike protein (HexaPro) expression construct sequence (DOI: 10.2210/pdb6XKL/pdb) was aligned with the sequence of the spike protein (mC-Spike) in the OMV-mC-Spike vaccine and the Spike protein (Spike) that was mixed with OMVs (OMV+Spike) in the mouse study. Both Spike and mC-Spike protein contain the D614G mutation not present in HexaPro. The mC-Spike protein contains a 3XGGGS repeat and a short amphipathic peptide, mCramp (GenBank accession number: CAA64078), an LPS-binding tag which are lacking in the Spike protein and HexaPro. (**B**) Native Page. The purified mC-Spike protein was loaded in lane 3. Three bands are visible. One strong band is visible at approximately 1000 kDa, a second strong band is visible at approximately 720 kDa and a faint band is visible at approximately 400 kDa. The strong bands of higher molecular weight are also visible at the same height in the reference material (trimeric, LuBio Science; lane 2). The three different bands most likely represent different oligomeric states of the trimeric spike protein. Based on experiments performed with a S-protein affinity resin that only binds the accessible (and hence active) receptor binding domain (RBD) of the Spike protein, it is assumed that the 720 kDa band is the species which is of major interest for this project. The band around 1000 kDa probably represents HMW species originating form S-protein instability (e.g. oligomeric form, aggregates). (**C**) SDS Page. 1 μg of purified mC-Spike protein was loaded on a non-reducing SDS-PAGE (lane 2). For reference, 1 μg of a commercial SARS-CoV-2 S-Protein (trimeric, LuBio Science; lane 1) showed a sharp and clearly defined band between 120 kDa and 200 kDa, as expected for the monomeric S-Protein. Some additional bands of low intensity were observed in the higher molecular area, possibly indicating oligomers and/or aggregates (trimer, ~400 kDa). No bands at low molecular weight were visible on the gel, indicating a not fragmented and pure Spike protein after purification. Compared to the reference material no significant difference was reported. (**D**) Dot blot showing association of pertactin to OMVs through linker mCRAMP or LL37. The virulence factor Pertactin (PRN), from *Bordetella pertussis*, was coupled to human antimicrobial peptide LL-37, or the murine variant thereof, called mCRAMP. It is expected that the coupled peptide will cause PRN to bind to OMVs after simply mixing them. As control proteins, PRN on its own and PRN linked to a scrambled version of mCRAMP, which should not bind to OMVs, were used. All proteins are provided with a His tag and produced as a recombinant protein. The OMVs used are from *Neisseria meningitidis* (ΔPorB ΔRmpM ΔlpxL1 Δcps). *Dot blot procedure:* Two 1.5 µl dots were placed on four cut-out pieces of nitrocellulose. One dot of either PRN, PRN-LL37, PRN-mCRAMP or PRN-scrambled mCRAMP, and one dot of OMV. Subsequently, the nitrocellulose pieces were washed with three times with 1 ml of Wst buffer (0.1 M Tris, 1.54 M NaCl, 5% Tween-80, pH 7.4) for five minutes. Next, the nictrocellulose pieces with OMV were incubated with 5 μl of the same protein as in the first dot. The staining procedure consisted of: washing with Wst buffer, incubation with anti-his Ab in Wst buffer, washing with Wst buffer, incubation with anti-mouse IgG-AP in wst-0.5%, washing with Wst buffer, washing with MiliQ, incubation with AP mix and wash again with MiliQ.

**
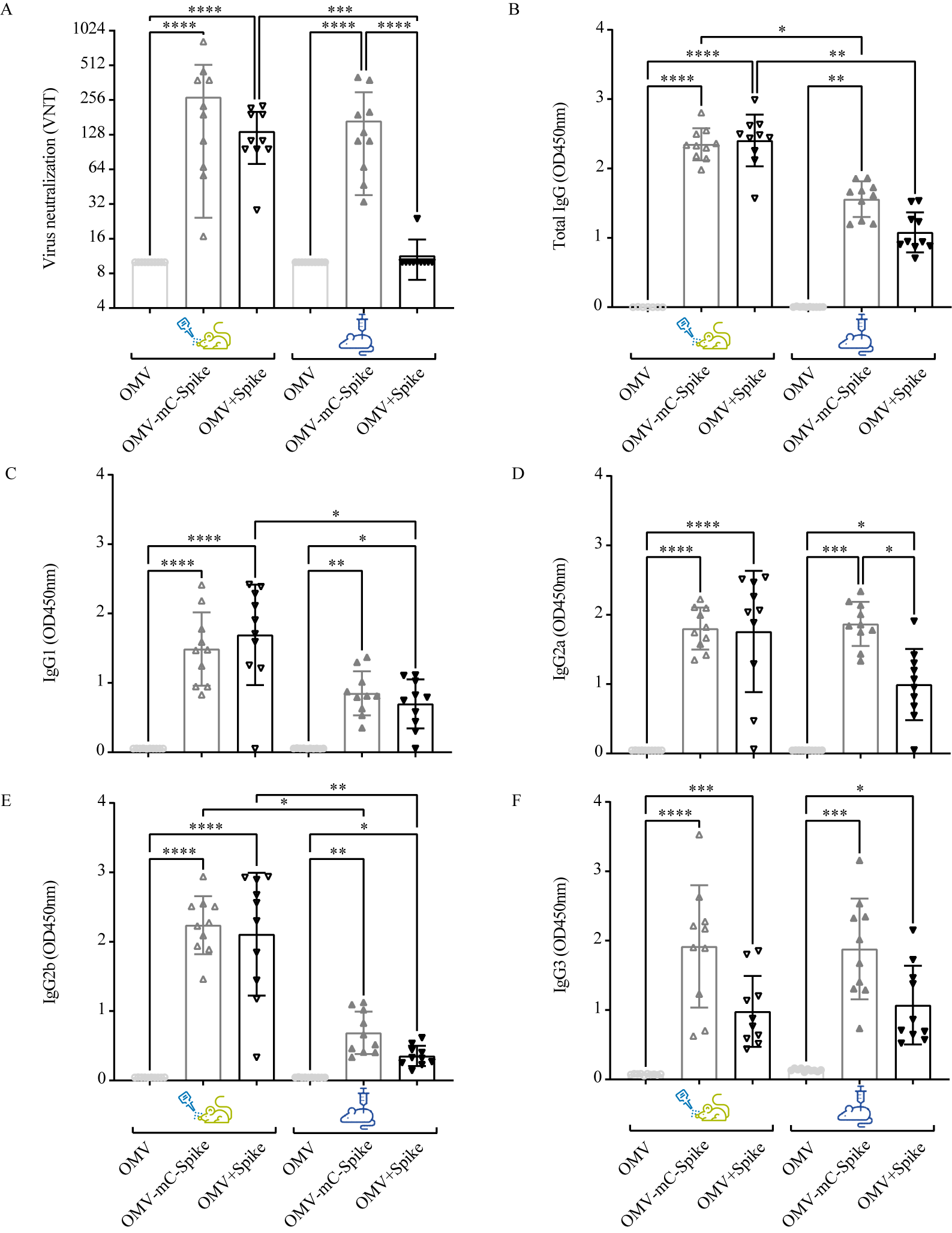
**

**Figure S2. Induction of virus neutralizing antibody titers and spike IgG antibody responses in mice.** Balb/C mice were immunized intranasally or intramuscularly on day 0 and day 21 with 15µg OMV (control group) or 15 µg OMV combined with 15µg Spike with mCRAMP (OMV-mC-Spike) or without mCRAMP (OMV+Spike). Sera was collected at day 35 and virus neutralizing titers (**A**) were determined. Furthermore spike IgG antibody levels were measured at day 35 with ELISA. Total IgG antibody levels were at 1:50000 (IgG1), 1:10000 (IgG2a), 1:50000 (IgG2b) and 1:100 (IgG3). Statistical significance was determined using the Kruskal-Wallis test followed by Two-stage linear step-up procedure of Benjamini, Krieger and Yekutieli multiple-comparison test. Significance is depicted as *p<0.05, **p<0.01***p < 0.001, **** p<0.0001.

**
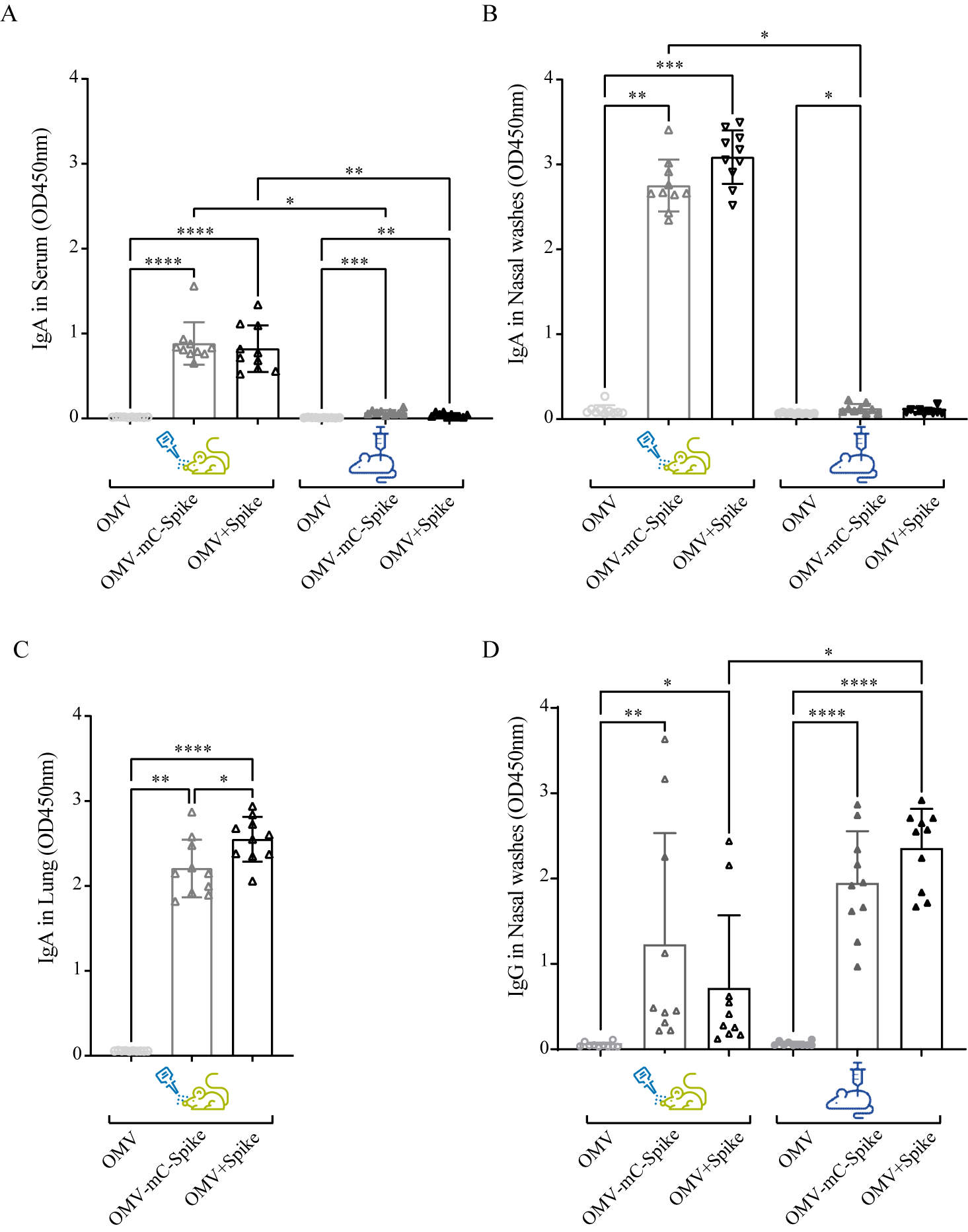
**

**Figure S3. IgA antibody responses after intranasal immunization in mice.** Balb/C mice were immunized intranasally or intramuscularly on day 0 and day 21 with 15µg OMV (control group) or 15 mg OMV combined with 15 mg Spike with mCRAMP (OMV-mC-Spike) or without mCRAMP (OMV+Spike). At day 35 sera and nasal washes were collected from all animals, and lungs were collected from animals which were intranasally immunized. IgA antibodies were measured with an ELISA in serum (1:200 dilution) (**A** ), nasal wash (1:1 dilution) (**B**) and lung (1:50 dilution) (**C**). IgG antibodies were measured in nasal washes (1:1 dilution) (**D**) Statistical significance was determined using the Kruskal-Wallis test followed by a two-stage linear step-up procedure of Benjamini, Krieger and Yekutieli multiple-comparison test. Significance is depicted as *p<0.05, **p<0.01***p < 0.001, **** p<0.0001.


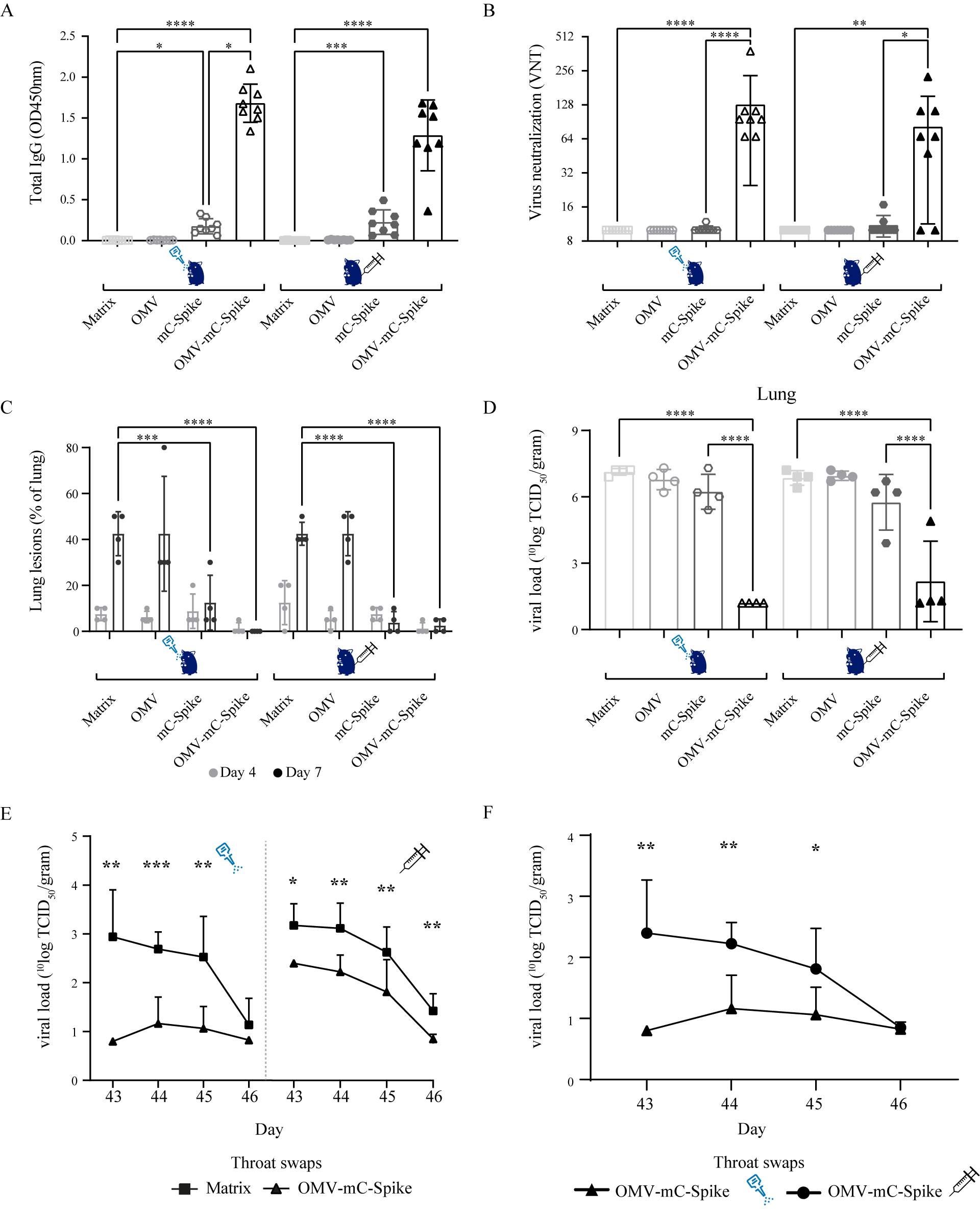


**Figure S4. Vaccination with OMV-mC-Spike protects against SARS-CoV-2 in Syrian hamsters.** Animals were immunized intranasally or intramuscularly on day 0 and day 21 with 15µg OMV, 15µg spike with mCRAMP (mC-Spike) or 15µg OMV combined with 15µg mC-Spike (OMC-mC-Spike). In the control group animals were immunized with 10mM Tris-3% sucrose, which is the OMV buffer. Sera were collected from all hamsters at experimental day 0, 21, 42, 46 and 49. At day 46 half of the animals per group (4 out of 8) were sacrificed and at day 49 the remaining 4 animals were sacrificed. (**A**) From the sera at day 42 total spike IgG antibodies were determined with an ELISA. Sera was 1:4000 diluted. (**B**) Virus neutralizing titers were determined in sera from day 42. (**C**) When animals were sacrificed at day 46 (day 4 post challenge) and day 49 (Day 7 post challenge) the percentage of the lung that presented lung lesions was quantified. The viral load was determined in lungs (**D**) and throat swabs (**E+F**). Statistical significance of the difference was evaluated by Kruskal-Wallis test and followed by a two-stage linear step-up procedure of Benjamini, Krieger and Yekutieli multiple-comparison test (**A, B** and **D**) or by a 2-way ANOVA test followed by Tukey’s multiple-comparison test (**C**), or by the non-parametric Mann whitney test (**E+F**). Significance is depicted as *p<0.05, **p<0.01***p < 0.001, **** p<0.0001.

**
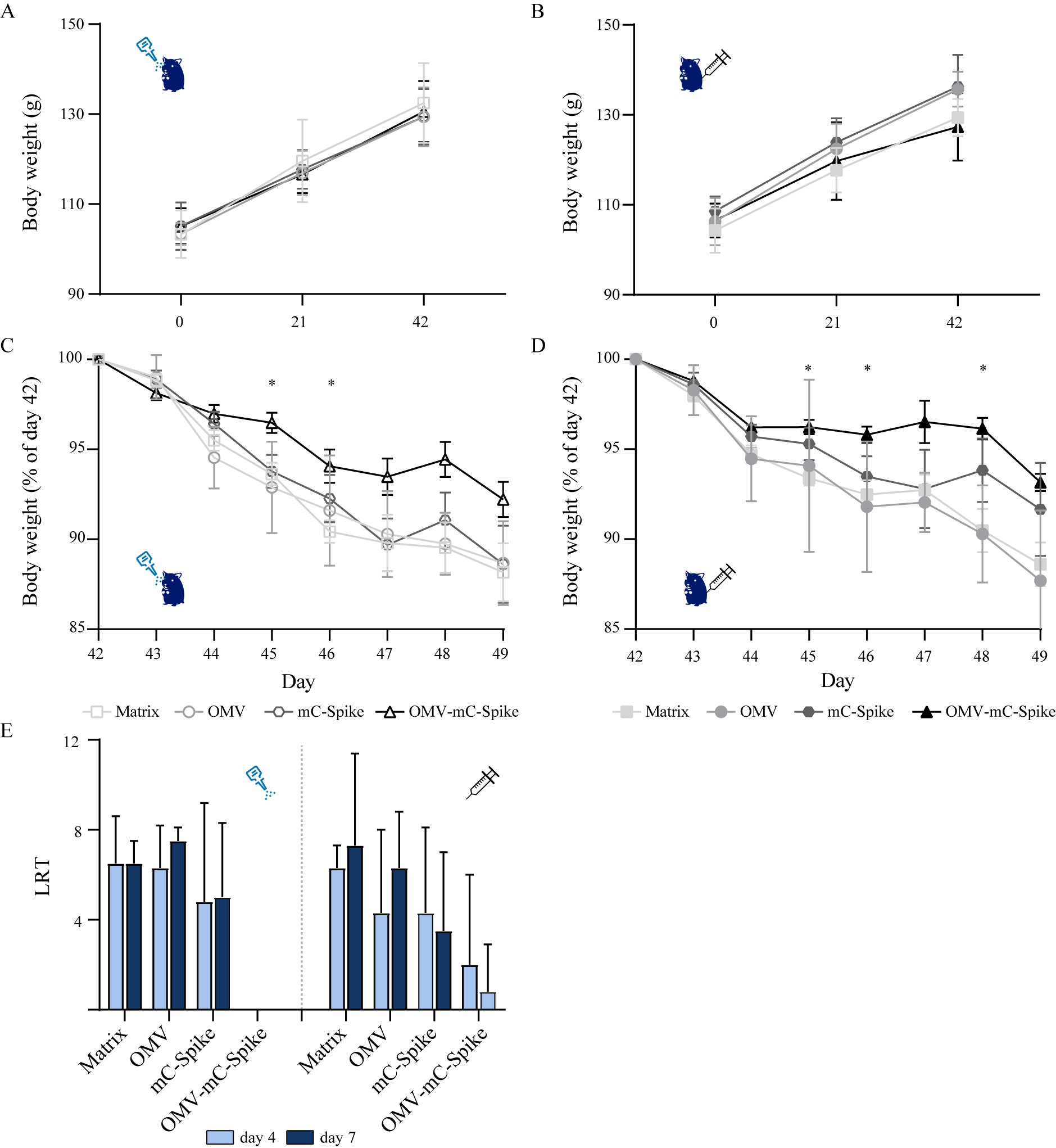
**

**Figure S5. Body weight and histopathological analysis.** Animals were immunized as described in supplementary 4. Bodyweight was measured at day 0, 21 and 42 in intranasal (**A**) and intramuscular (**B**) immunized animals prior to challenge with SARS-CoV-2. After challenge, the bodyweight was daily measured in intranasal (**C**) and intramuscular (**D**) immunized animals. Day 47-49 only include 4 animals, because half of the animals were sacrificed at day 46. Histopathological analysis from lung tissues was performed for all animals. Lung tissue was analyzed and scored for presence and severity of alveolitis, alveolar damage, alveolar edema, alveolar hemorrhage, type II pneumocyte hyperplasia, bronchitis, bronchiolitis, peribronchial and perivascular cuffing. Scores are presented as sum of Lower Respiratory Tract (LRT) disease parameters. Statistical significance was determined with a 2-way ANOVA test followed by Tukey’s multiple-comparison. Significance is depicted as *p<0.05, **p<0.01***p < 0.001, **** p<0.0001.
